# Influence of Risk Factors for Male Infertility on Sperm Protein Composition

**DOI:** 10.3390/ijms222313164

**Published:** 2021-12-06

**Authors:** Marie Bisconti, Jean-François Simon, Sarah Grassi, Baptiste Leroy, Baptiste Martinet, Vanessa Arcolia, Vladimir Isachenko, Elise Hennebert

**Affiliations:** 1 Laboratory of Cell Biology, Research Institute for Biosciences, University of Mons, Place du Parc 20, 7000 Mons, Belgium; marie.bisconti@umons.ac.be (M.B.); sarah.grassi@student.umons.ac.be (S.G.); elise.hennebert@umons.ac.be (E.H.); 2 Fertility Clinic, CHU Ambroise Paré Hospital, Boulevard Kennedy 2, 7000 Mons, Belgium; jean-francois.simon@hap.be (J.-F.S.); vanessa.Arcolia@hap.be (V.A.); 3 Laboratory of Proteomics and Microbiology, CISMa, Research Institute for Biosciences, University of Mons, 7000 Mons, Belgium; baptiste.leroy@umons.ac.be; 4 Evolutionary Biology & Ecology, Université Libre de Bruxelles, Avenue Paul Héger, CP 160/12, 1000 Brussels, Belgium; Baptiste.martinet@ulb.be; 5 Department of Obstetrics and Gynecology, University of Cologne, Kerpener Strasse 34, 50931 Cologne, Germany

**Keywords:** infertility risk factors, sperm, proteins, proteomics, obesity, diabetes, tobacco smoking, bisphenol-A

## Abstract

Male infertility is a common health problem that can be influenced by a host of lifestyle risk factors such as environment, nutrition, smoking, stress, and endocrine disruptors. These effects have been largely demonstrated on sperm parameters (e.g., motility, numeration, vitality, DNA integrity). In addition, several studies showed the deregulation of sperm proteins in relation to some of these factors. This review inventories the literature related to the identification of sperm proteins showing abundance variations in response to the four risk factors for male infertility that are the most investigated in this context: obesity, diabetes, tobacco smoking, and exposure to bisphenol-A (BPA). First, we provide an overview of the techniques used to identify deregulated proteins. Then, we summarise the main results obtained in the different studies and provide a compiled list of deregulated proteins in relation to each risk factor. Gene ontology analysis of these deregulated proteins shows that oxidative stress and immune and inflammatory responses are common mechanisms involved in sperm alterations encountered in relation to the risk factors.

## 1. Introduction 

Spermatozoa are highly specialised and differentiated cells that form from spermatogonial stem cells (SSCs) during spermatogenesis in the seminiferous tubules of the testicles [1]. Disruption of spermatogenesis or post-testicular events can lead to fertility disorders [2]. Infertility is a common health problem defined as the inability of a couple to achieve a spontaneous pregnancy after a year or more of unprotected sex. Two types of infertility can be distinguished: primary infertility, for couples who have never conceived a child, and secondary infertility, for couples who have already achieved a spontaneous pregnancy but are unable to conceive again [3]. Infertility disorders affect about 15% of couples [4] and impaired sperm quality appears to be involved in about 50% of cases [5,6].

With reproductive history, semen quality analyses are the primary steps in the assessment of male infertility [4,7]. These analyses include the determination of ejaculate volume, pH, viscosity, and appearance as well as sperm count, motility, vitality, and morphology, whose values are compared to reference values established by the World Health Organisation (WHO) laboratory manual for the examination and processing of human semen [8]. However, in over 30% of cases, this routine semen examination is insufficient to explain a man’s infertility [9]. Other diagnostic tests can be performed to investigate male fertility disorders but are not routinely used in fertility clinics. These tests aim at determining the ability of sperm to undergo capacitation, acrosome status, membrane integrity, interaction with the oocyte, mitochondrial activity, DNA integrity, etc. [10]. Furthermore, an increasing number of studies focus on the molecular mechanisms involved in male infertility (see [11,12,13,14], for reviews). Among them, proteomic approaches have been widely used to identify key proteins involved in infertility disorders, mainly by comparing sperm samples from different donors (e.g., normospermic samples from fertile vs. infertile men, asthenospermic vs. normospermic sperm, etc.). The results of these studies, focused on altered sperm parameters and some diseases, have already been compiled in excellent reviews (e.g., [15,16,17,18,19,20]).

Several studies also focused on the detrimental effects of risk factors for male infertility on the protein composition of sperm. Indeed, in addition to diseases, more and more factors are highlighted for their negative impact on male reproductive potential [21,22]. Among these infertility risk factors, the most common is age: although men are fertile throughout their lives, sperm parameters decline from the age 35 [23]. In addition, diet, caffeine intake, weight, physical activity, psychological impact (e.g., stress), smoking, drug or alcohol use, medication, diabetes, exposition to synthetic chemicals, clothing, sleep, etc., can cause fertility impairment [22,24,25,26,27,28]. Although these risk factors linked to male infertility are very numerous, only a few of them have been the focus of sperm proteomic analyses. In this review, we have inventoried the literature related to the identification of sperm proteins showing abundance variations in response to the four risk factors for male infertility the most investigated in this context: obesity, diabetes, tobacco smoking, and exposure to bisphenol-A (BPA). We present an overview of the techniques used to identify deregulated proteins and we summarise the main results obtained in the literature for each risk factor. Then, we provide a compiled list of the deregulated proteins that we submit to Gene Ontology (GO) analysis to highlight the biological processes in which they are involved.

## 2. Methods

An exhaustive literature search was conducted on Google Scholar and PubMed by using the terms “sperm/spermatozoa/male fertility + risk factor + protein/proteome” to identify articles related to sperm proteins and the above-mentioned risk factors for infertility, published in English. All the studies included were performed on either the whole semen, the seminal plasma, the complete set of cells in the semen, or the purified spermatozoa from human, mouse, or rat (Table 1). Although several studies were performed on testis and epididymis, we decided to exclude them to avoid deregulated proteins that could be derived from cell types other than sperm. As the purpose of this review was to constitute a list of sperm proteins deregulated by each risk factor, we considered only studies focusing on the risk factor itself, trying to avoid the synergic effects caused by other clinical disorders. For instance, we did not include in this review studies on smoking men with varicocele (e.g., [29,30]). Moreover, only studies focusing on changes at the protein level were considered for this review, thereby excluding RNA and post-translational modification analyses. 

A list of all the deregulated proteins identified in the studies was compiled (Table S1). To establish this list, we annotated the proteins using the UniProtKB accession numbers, even if these numbers were not indicated in the original articles. Some of the UniProtKB accession numbers cited in the studies had become obsolete and therefore, we replaced them with the correct accession number or with “N/A” if the protein could not be found in the database. In some cases, the protein name from the articles did not match the UniProtKB accession number provided in the article, so we chose the updated protein name and wrote “renamed as” in the compiled list. In studies based on separation by gel electrophoresis (2D-PAGE), some proteins were identified several times in different gel spots. In our list, we included these proteins only once. In the original studies, some proteins possessed different names, but were found to correspond to the same UniProtKB accession number. Again, we kept these proteins only once. In the studies focusing on specific proteins, the authors did not always specify the targeted isoform or subunit. In these cases, we chose to provide the accession number of the isoform/subunit that would best fit the study in question. These proteins appear as “selected” in Table S1. Finally, we also integrated studies focussing on the activity of some enzymes, rather than their abundance. In these cases, “activity” is specified together with the upregulation or downregulation in Table S1. Then, based on this compiled list, we generated a list of proteins that appear to be statistically and biologically deregulated for each risk factor. For this, the list of proteins identified by proteomic profiling (see Section 3.2) was manually curated by (1) removing the proteins for which we did not find any Uniprot ID, and (2) keeping only the proteins for which a fold change ≥1.5 or ≤0.66 was measured between the tested groups (Table S2). Finally, we created sublists separated for human and rodent (in which we removed redundant proteins between the studies and protein isoforms translated from the same gene) that were analysed using the bioinformatics tool DAVID v6.8 (Database for Annotation, Visualisation and Integrated Discovery; http://david.abcc.ncifcrf.gov/ accessed on 27 September 2021) in order to identify overrepresented Gene Ontology (GO) Biological Process terms [44,45]. The results were considered statistically significant if *p* < 0.05.

## 3. Experimental Strategies for Identification of Sperm Proteins Deregulated by Risk Factors

Two main approaches have been used to identify and quantify sperm proteins impacted by infertility risk factors: (1) the analysis of all the proteins contained in a sample by mass spectrometry, called “proteomics”, and (2) the detection of a few target proteins of already known function. In both cases, proteins from the investigated group (e.g., smokers) were compared to those from the control group (e.g., non-smokers).

### 3.1. Sample Preparation

**Figure 1:**
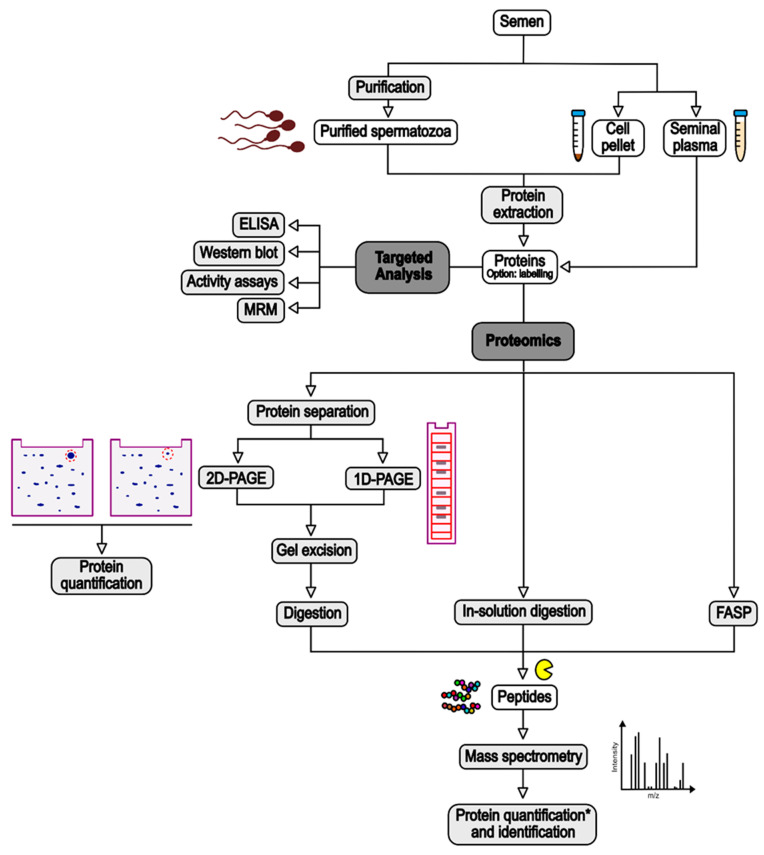
Summary of experimental strategies used in the studies cited in this review for the identification of sperm proteins deregulated by risk factors. See text for detailed explanations. ELISA: Enzyme-Linked ImmunoSorbent Assay; MRM: Multiple-Reaction Monitoring. *: Quantification based on MS signal is for proteins excised from 1D-PAGE or label-free strategies, while quantification for 2D-PAGE is based on the intensity of gel spots.

Different samples can be used to investigate sperm proteins: the seminal plasma or the complete set of cells in the semen, recovered as the supernatant and the pellet after centrifugation of sperm (e.g., [31,32]), respectively; or purified spermatozoa, isolated by swim-up (e.g., [39]) or density gradient (e.g., [33]) (Figure 1). The purpose of this purification is to eliminate contaminating cells such as epithelial cells, leukocytes, and cells from early stages of spermatogenesis, whose protein content could decrease the capacity to discriminate deregulated proteins in spermatozoa [20,46]. However, some recent studies showed that the influence of round cell and leukocyte proteins was not significant [47,48]. While seminal plasma is composed of soluble proteins which can be analysed almost directly, sperm cells have to be lysed to extract the proteins (Figure 1). This is performed by incubation with a lysis buffer with or without sonication. The composition of the lysis buffer depends on the technique used to process the extracted proteins. Usually, it contains chaotropic agents (e.g., urea), reducing agents (e.g., dithiotreitol—DTT), and detergents, which can be ionic (e.g., sodium dodecyl sulphate—SDS), non-ionic (e.g., Triton-X-100), or zwitterionic (e.g., 3-[(3-cholamidopropyl)dimethylammonio]-1-propanesulfonate—CHAPS) (e.g., [32,34,39]). Although SDS is a highly effective solubilising agent, this ionic detergent causes a background signal in MS analyses, leading to the suppression of the peptide signal [49]. Thus, it must be removed from the samples prior to MS analysis. This can be executed by protein precipitation with organic solvents or with column-based approaches [50]. In sperm proteomics, filter-aided sample preparation (FASP), in which SDS is removed and exchanged with urea using a filtration device, has become a popular technique (e.g., [35,36]) (Figure 1). In most of the studies, extracted proteins are then quantified using a colorimetric protein assay such as bicinchoninic acid (BCA) or Bradford (e.g., [31,39]).

### 3.2. Proteomics

Mass spectrometry (MS) is a sensitive and robust tool that can monitor the abondance of hundreds or thousands of proteins at once [51]. Table 1 summarises proteomic analyses performed to identify regulated proteins in relation to risk factors for fertility and used in this review.

Before processing for MS, extracted proteins can be submitted to a step of labelling to perform ulterior protein quantification. Labelling can be conducted with various methods, reviewed in [52]. In the studies integrated in the present review, protein labelling was performed through the covalent attachment of fluorescent dyes such as Cy3 or Cy5 (e.g., [33]) or of isotope-labelled molecules (e.g., isobaric tag for relative and absolute quantitation—iTRAQ, [38]). However, most of the studies cited in this review used a label-free method for protein quantification (e.g., [31,35,37,40]) (Figure 1). 

After this step, proteins are submitted to enzymatic digestion, often by trypsin, to generate peptides that will be analysed by MS. Protein digestion can be performed directly on the proteins (“in-solution digestion”), on the filter in the case of FASP, or after protein separation by one- or two-dimensional polyacrylamide gel electrophoresis (1D- or 2D-PAGE) (e.g., [32,34,41]) (Figure 1). In the latter case, to allow for protein quantification, the gels are scanned with a biomolecular imager, either directly in the case of Difference Gel Electrophoresis (DIGE) of fluorescently labelled proteins (e.g., [33]), or after staining for conventional PAGE (e.g., [41]). Protein spots are quantified based on the signal intensity, and proteins of interest (i.e., with different intensity between the study and control groups) are excised from the gels before submission to trypsinolysis (Figure 1).

Tryptic peptides are then analysed with a mass spectrometer, which detects peptides based on their mass-to-charge ratio (*m*/*z*) [51] (Figure 1). Often, the peptides are separated onto an analytical column according to their hydrophobicity prior to their MS analysis, in order to reduce the complexity of the peptide mixture. This is called liquid chromatography (LC)-MS/MS (e.g., [38,40]). The peptides are then ionised by matrix-assisted laser desorption/ionisation (MALDI) or electrospray ionisation mass spectrometry (ESI) and analysed by the mass spectrometer. Different types of mass spectrometer exist and have been reviewed in [51]. They are not discussed in this review. Acquired MS/MS data are then compared to protein databases from the investigated species to identify the proteins present in the samples. This comparison step is performed following different criteria which ensure the confidence in protein identification and limit the presence of false discovery results [53].

To identify differentially expressed proteins between the study and the control groups, different strategies are used (Figure 1). In the case of proteins previously separated by electrophoresis, the quantification is performed upstream of the protein digestion. An abundance ratio, also called fold-change, is then calculated based on the differential intensity of the protein spots between the groups. In the case of in-solution digestion, fold changes are calculated based on the detection of the tags added to the proteins (e.g., iTRAQ) or inferred from MS or MS/MS-derived signals (e.g., [31,32,35]). Replicated experiments allow statistical analyses of the data to establish a *p*-value associated with the calculated fold change. In addition to this *p*-value, exclusion criteria are applied (e.g., only fold changes above or below defined levels are considered) to increase the confidence in the results [54]. 

Although MS analyses based on 2D protein separation have been used for many proteomic studies, it is well known that gel-free approaches combined with LC-MS/MS, called “shotgun proteomics”, are more sensitive and allow the identification of a significantly higher number of proteins [52].

### 3.3. Targeted Analyses

Several tests involve the use of antibodies directed against specific proteins to investigate their abundance variations between different tested groups (Figure 1). The most common are certainly Enzyme-Linked ImmunoSorbent Assay (ELISA; [55,56]) and Western blotting (e.g., [57]). In ELISA, the target protein is immobilised on the well surface of a microplate via capture by a specific antibody, and then probed with another antibody linked to a reporter enzyme or detected with a secondary antibody linked to the reporter enzyme. After incubation with the enzyme substrate, the resulting by-product is quantified [58]. In Western blotting, proteins are separated by SDS-PAGE and transferred to a solid support, generally a nitrocellulose or a Polyvinylidene Fluoride (PVDF) membrane. The membrane in then incubated into the primary antibodies targeting the protein of interest, followed by an incubation in a secondary antibody which can be visualised by various methods such as chemiluminescence or immunofluorescence. The abundance of the protein is then inferred from the intensity of the labelled protein band [59].

Recently, our team used Multiple-Reaction Monitoring (MRM) to investigate the abundance of HSP70 isoforms in spermatozoa (unpublished results, see Section 4.1). MRM is a very robust and sensitive mass spectrometry method allowing targeted analysis of peptides and proteins of interest [60].

All the cited methods can also be used to validate the abundance variations of specific proteins previously highlighted by untargeted proteomic workflows. Western blotting has been widely used in the studies integrated in this review for such validation, but MRM-based validation strategies will certainly be used more and more in the future. In addition to these methods, different quantification assays have been used to study the variation of the activity of different enzymes (e.g., [61,62,63,64,65]).

## 4. Impact of Infertility Risk Factors on Sperm Proteins

The impact of obesity, diabetes, tobacco smoking, and exposure to BPA on sperm proteins has been studied in human and rodent models. Some studies used a global proteomic approach to identify the deregulated proteins while others focused on target proteins.

### 4.1. Obesity

According to the WHO, overweight and obesity correspond to abnormal or excessive fat accumulation. These metabolic disorders are influenced by various environmental, hormonal, and genetic factors and are often determined using body mass index (BMI) data. Indeed, people affected by overweight and obesity have a BMI ≥ 25 kg/m² or ≥ 30 kg/m², respectively [66]. Reduction of sperm quality has been observed in obese men compared to those with normal weight. Although it remains controversial, lower semen volume, decreased sperm concentration, motility and morphology, and increased sperm DNA damage may be correlated with obesity [67,68,69,70,71,72]. These effects on sperm parameters have been attributed to defective spermatogenesis caused by a deregulation of the hypothalamic–pituitary–gonadal axis, chronic inflammation, increased testicular heat, and oxidative stress [70,73,74].

In order to identify obesity-associated proteomic changes in spermatozoa, Kriegel et al. [33] used 2D-DIGE to compare the proteome of progressive normomorphic spermatozoa from five normospermic clinically healthy donors and two non-diabetic obese patients (Table 1). Using MALDI-TOF-MS, they identified nine different proteins apparently associated with obesity. One of them, outer dense fibre protein 1 (ODF1), a structural protein of the sperm tail, was found in several gel spots, reflecting that the protein was present as multiple molecular forms resulting from proteolysis and/or post-translational modifications [33]. According to the gel spot, ODF1 was either more or less expressed in obese patients in comparison to the healthy donors. Regarding the eight other proteins, two were more expressed and six were less expressed in obese patients (Table S1). The spermatogenic glyceraldehyde 3-phosphate dehydrogenase (GAPDHS), a sperm-specific glycolytic enzyme which has been shown to be involved in sperm motility [75], was particularly abundant in obese men, with a tenfold increase [33]. Later on, the same team extended its study to a larger cohort of individuals. Using the same experimental design, the authors compared 21 normospermic healthy donors and 13 non-diabetic obese patients [34]. In that case, they identified seven proteins differentially expressed between the two groups, all with increased levels in obese patients, except for 1, ß-Galactosidase-1-like protein (GLB1L), which was reduced (Table S1). On the six increased proteins, two, lactotransferrin (LTF) and semelogenin-1 (SEMG1), were present in different gel spots, implying their occurrence as different molecular forms [34]. Notably, the set of proteins observed as deregulated in this second study was different from the one found in the previous study, highlighting the necessity to use a fairly large number of patients in such studies. Among the proteins present in higher levels in obese patients, the authors noted that three proteins—clusterin (CLU), LTF, and SEMG1—are bound to eppin (epididymal protein inhibitor) in the so-called eppin protein complex (EPC) [34]. EPC is present in human seminal plasma and on the surface of the tail of ejaculated spermatozoa and has been proposed to be involved in protection of spermatozoa and regulation of motility [76]. Paasch et al. [34] proposed that the increased levels of modified forms of LTF and SEMG1 found in obese patients could be involved in the alteration of sperm functions in these individuals.

Recently, Pini et al. [37] studied the impact of obesity on sperm proteome by using LC-MS/MS analysis and label-free quantification. They included in their study only obese men and men with a healthy weight presenting normal sperm parameters and excluded patients smoking tobacco or with clinical conditions (e.g., diabetes, hypertension) to limit the influence of other risk factors. By comparing samples from five individuals in each group, they identified 24 proteins which were less abundant and three proteins which were more abundant in the spermatozoa from obese men than in the control group (Table 1 and Table S1). The validity of the MS results was confirmed on a new cohort of patients on four selected proteins using immunofluorescence and/or quantitative protein immunoassay [37]. Based on a literature search, the authors showed that 14 of the differentially expressed proteins could be classified into different categories of biological processes: oxidative stress, inflammation, translation, DNA damage and sperm function (acrosome reaction, motility, nucleus stabilisation). They hypothesised that inflammation and stress responses induced by obesity impact gene expression during spermatogenesis, thereby causing sperm function alteration [37].

Another study focused on proteomic changes associated with obesity-induced asthenospermia. Using a label-free quantitative LC-MS/MS approach, they compared the proteome of spermatozoa from three obese individuals with severe asthenospermia and three clinically healthy individuals [35] (Table 1). They identified 127 proteins which were deregulated in the obesity-associated asthenospermic group in comparison to the control group, including 105 less abundant proteins and 22 more abundant proteins (Table S1). As expected, some of these proteins were identified in other proteomic studies comparing normospermic and asthenospermic samples [77,78]. Using gene ontology (GO) analysis, the authors showed that the differentially expressed proteins were mainly involved in protein metabolism, cellular component biogenesis and assembly, translation, and vesicle localisation and targeting [35]. In addition to this MS discovery analysis, the authors confirmed the downregulation of two proteins, endoplasmic reticulum protein 57 (ERp57) and actin-binding-related protein T2 (ACTRT2), in a new cohort of obesity-associated asthenospermic individuals by immunofluorescence, flow cytometry and Western blot. They showed for the first time an implication of these two proteins in sperm motility [35].

Seminal plasma has also been investigated to identify changes in protein abundance associated with obesity in humans. Ferigolo et al. [31] investigated the seminal plasma from 27 obese men and 20 men with a healthy weight, excluding individuals with clinical conditions that could affect fertility (Table 1). They showed that 70 proteins were differentially expressed between the two groups: 1 was found only in the seminal plasma from obese men, 50 were over-represented, and the 19 others were under-represented in the obese group [31] (Table S1). Using functional enrichment analysis, the authors showed that several functions were enriched in the seminal plasma of obese men in comparison to the control men, including apoptosis pathway regulation, inflammatory and immune responses, and antioxidant activity [31]. 

Some studies used a rodent model to assess the effect of obesity on sperm proteome. To this end, they compared mice or rats fed with a control diet (CD group) and mice or rats fed with a high-fat diet (HFD group) for several weeks. Peng et al. [36] compared the proteome of spermatozoa from six CD mice and six HFD mice using a label-free LC-MS/MS analysis (Table 1). They identified 160 proteins differentially regulated between the two groups, of which 100 were less abundant and 60 were more abundant in the HFD group (Table S1). GO analysis classified most of the differentially expressed proteins in the following biological processes: transport, intracellular protein traffic, cell structure and motility, exocytosis, and endocytosis [36]. Among the down-regulated proteins, the authors specifically focused on centrosome and spindle pole associated protein 1 (CSPP1) and centrin 1 (CETN1), two cytoskeletal-related proteins, and confirmed their lower abundance in HFD group by Western blotting. The same conclusion was reached in humans by comparing centrifuged sperm from normal men to obese and overweight men [36]. The authors suggested that obesity-induced down-regulation of these proteins during spermatogenesis could explain altered sperm morphology in obese males [36]. More recently, Carvalho et al. [32] studied the proteome of spermatozoa from 10 rats from each group by analysing protein bands after SDS-PAGE by LC-MS/MS (Table 1). Based on the calculation of the exponentially modified protein abundance index (emPAI) and statistical analyses, they highlighted 15 proteins whose abundance varied between the CD and HFD rats, eight more abundant and seven less abundant in HFD (Table S1). These proteins were structural proteins or proteins involved in cellular respiration, metabolic activity and protection [32]. 

In addition to these large-scale proteomic studies, some studies focused on specific proteins to assess the impact of obesity on male fertility (Table S1). Shi et al. [61] showed that the level and activity of protein-tyrosinase phosphatase 1B (PTP1B), a negative regulator of the leptin (LEP) and insulin (INS) signalling pathways [79], were significantly increased in obese mice and men and were correlated to alteration in sperm acrosome reaction. Using ELISA, Fan et al. [56] showed that interleukin-6 (IL-6) and tumour necrosis factor (TNF-α), two inflammatory markers, were increased in the seminal plasma of obese patients. Together with altered semen quality and the presence of increased levels of signal factors regulated by TNF-α in the testis, these results showed the importance of chronic inflammation of male genital tract in the fertility of obese individuals [56]. Two protein hormones involved in the modulation of the hypothalamus–pituitary–testes (HPT) axis [80], LEP and INS, were also shown to be present at increased levels in the seminal fluid of obese men [81] and in the seminal vesicle fluid of obese mice [82]. Recently, using MRM-MS, our team investigated the relative abundance of the isoforms of the chaperon protein HSP70 in spermatozoa from 17 normospermic men with a BMI ranging between 18.4 and 38.3 kg/m^2^. Although no significant correlation could be established between the BMI and the abundance of HSP70 isoforms, our results showed a trend of increased abundance for HSPA1L and HSPA8 according to the BMI, and a trend of decreased abundance for HSPA5 (Figure S1, unpublished results).

Based on the criteria defined in the Methods section, we obtained a list of 388 proteins deregulated in cases of obesity (Table S2). Among these, only 11 (2.8%) were identified in at least two independent studies, and for some of them, opposite variations were observed (Table S2). This was not unexpected, considering the high variability into the different studies regarding the species (human, rat or mouse), the samples (seminal plasma or spermatozoa, those being purified or not), the group considered (normomorphic obese or asthenospermic obese), and the identification method (2D-PAGE or gel free, different mass spectrometers) used (Table 1). Among the common proteins highlighted in the different studies, several are already known for their important role in sperm function and their deregulation could therefore explain fertility troubles encountered in cases of obesity. GADPHS was deregulated in three of the up-cited studies, although with different variations. Liu et al. [35] showed a decrease in the abundance of this glycolytic enzyme in cases of obesity while Kriegel et al. [33] and Carvalho et al. [32] showed an upregulation in the obese group. This discrepancy can be explained by the fact that Liu et al. [35] investigated obese patients with severe asthenospermia while the two other studies investigated normospermic obese groups which did not present alteration of sperm motility. Indeed, it has been shown that decreased expression of GAPDHS leads to defects in sperm motility [75,83]. SEMG1 is a seminal protein which has been shown to inhibit premature sperm capacitation [84]. Its decrease in the sperm of obese patients [33,34] could therefore cause reduced fertility. Phospholipase A2 (PLA2B)-decreased expression was highlighted in the seminal plasma [31] and purified spermatozoa from obese patients [35]. This seminal enzyme is associated to the sperm plasma membrane [85] and is involved in membrane fusion events occurring during the acrosome reaction and the fusion between the spermatozoon and the oocyte [86]. Its decreased abundance in the sperm of obese patients could therefore lead to fertilisation defects. Apolipoprotein A1 (ApoA1) is a sterol acceptor present in the seminal fluid [87] which has been shown to activate sperm motility [88]. In the up-cited studies, it presented opposite variations, with an increased abundance in the seminal plasma from obese men [31] and a decreased abundance in the purified spermatozoa from HFD mice [36]. Other interesting proteins are LEP and INS, which were found at increased levels in the seminal fluid of obese patients [81] and mice [82]. Interestingly, increased seminal LEP concentration has been reported to be correlated with decreased sperm motility [89].

**Figure 2:**
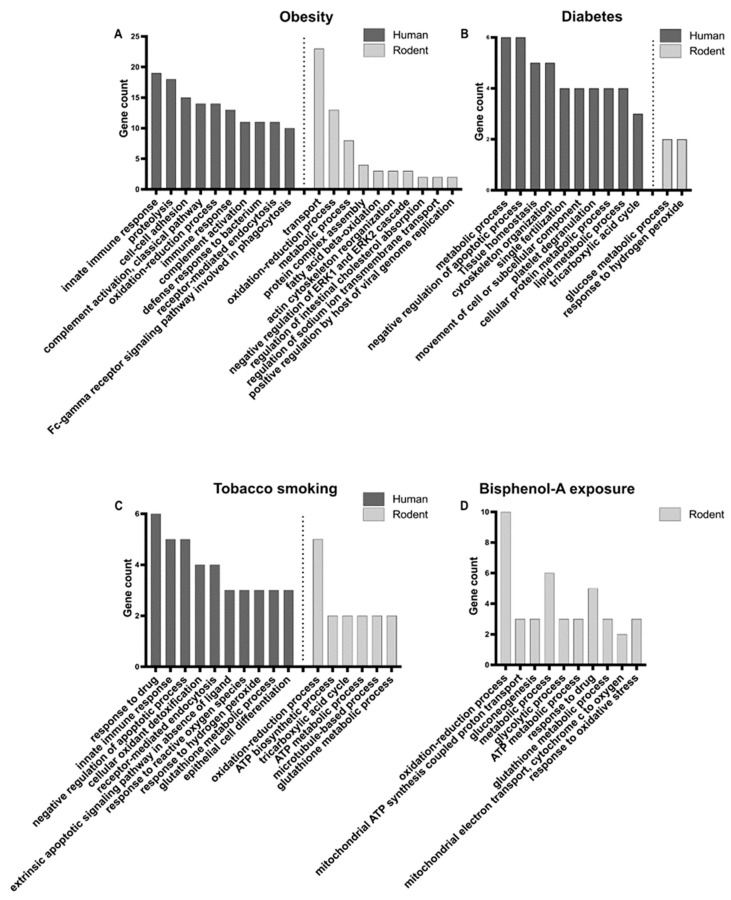
Enriched GO biological processes for deregulated sperm proteins in human and rodent models in relation to obesity (**A**), diabetes (**B**), tobacco smoking (**C**), and bisphenol-A (BPA) exposure (**D**). Top 10 enriched processes are presented. For BPA, only the protein list from mice was submitted to GO analysis. See Table S3 for complete dataset.

GO analyses were performed on the protein lists from human and rodents separately, in order to identify enriched biological processes (Figure 2A, Table S3). In human, top enriched biological processes were related to immune response, proteolysis, cell–cell adhesion, and the oxidation–reduction process, while in rodents they were mainly related to transport (i.e., movement of substances and cellular components), the oxidation–reduction process, and the metabolic process. Therefore, the oxidation–reduction process appears to be a common important mechanism deregulated in cases of obesity. Indeed, oxidative stress, caused by an imbalance between the production and neutralisation of reactive oxygen species (ROS) [90], has been shown to be responsible for spermatogenesis disturbance and alteration of sperm quality in obese men [70,73]. Higher levels of ROS appear to cause DNA damage, membrane oxidation, and disruption of mitochondrial activity in sperm [74,91]. The variation in abundance of antioxidant proteins could therefore act to mitigate the negative effects of ROS in obese men [92]. The immune response highlighted in humans is certainly linked to the chronic inflammatory status of the obese male genital tract, characterised by the abnormal production of cytokines (such as interleukins and tumour necrosis factors), which was shown to impair spermatogenesis in testicular tissues and sperm maturation in epididymis [56,68,71,74,93].

### 4.2. Diabetes

Diabetes is a chronic disease that occurs when INS, a hormone that regulates blood sugar, is not produced in sufficient quantities by the pancreas (type-1 diabetes) or when the body is unable to effectively sense the INS it produces (type-2 diabetes) [94]. The risk of infertility is higher in men with diabetes compared to men without diabetes [27,95]. Indeed, some studies showed that men with diabetes presented decreased sperm count and motility as well as increased sperm apoptosis and nuclear and mitochondrial DNA damage [27,96,97,98,99]. Diabetes-related fertility disorders could be associated to dysfunctional spermatogenesis caused by epigenetic dysregulation, oxidative stress, endocrine disorders, and diabetic neuropathy [95,99].

In addition to the impact of obesity, Kriegel et al. [33] also investigated the impact of diabetes on the sperm proteome. Analysis of the proteomic profile of progressive normomorphic spermatozoa from two type-1 diabetic patients and five normospermic donors by 2D-DIGE combined to MALDI-TOF-MS resulted in the identification of seven differentially expressed proteins: four more abundant in diabetic men, two less abundant, and one detected in three gel spots and whose relative abundance varied according to the gel spot (Table 1 and Table S1). In their subsequent study already described above in the case of the study of obesity, the research group compared the proteome of purified spermatozoa from 21 healthy individuals, eight type-1 diabetic patients, and seven type-2 diabetic patients [34] (Table 1). For type-1 diabetes, they identified six up-regulated proteins and two down-regulated proteins, while for type-2 diabetes, they identified 12 up-regulated proteins and 24 down-regulated proteins, again with some proteins present as different molecular forms that could result from post-translational modifications and/or proteolytic degradation [34] (Table S1). As in the obese group, the authors highlighted the increased levels of CLU, LTF, and SEMG1, components of the EPC, as well as of their modified forms, thereby implying an alteration of this complex in diabetic men [34].

Later, a study conducted by An et al. [38] used iTRAQ labelling and LC-MS/MS to compare the sperm proteome from six type-2 diabetic men and six healthy donors (Table 1). They identified 357 differentially expressed proteins, of which 38 were less abundant and 319 were more abundant in diabetic patients (Table S1). Western blot analyses validated the differential expression of four selected proteins [38]. Among the deregulated proteins, 14 were related to mitochondrial metabolism. These results, associated with the observation of altered mitochondrial ultrastructure in spermatozoa from obese men, led the authors to propose that alteration of sperm motility is a cause of diabetes-induced male infertility [38].

The impact of diabetes on the proteome of spermatozoa was also studied in rats by Carvalho et al. [32], including obese rats (see above) (Table 1). In this case, the rats were intraperitoneally injected with streptozotocin to induce diabetes [32]. Of the 15 deregulated proteins highlighted in this study (see Section 4.1 on obesity), three were more abundant and 12 were less abundant in the diabetic rats in comparison to the control group (Table S1). 

After applying our criteria defined in the Methods section, we obtained a compiled list of 82 proteins which appear to be deregulated in relation to diabetes, of which nine (11%) were common to two independent studies (Table S2). As observed for obesity, opposite variations in the abundance of some of these proteins were observed between studies. For instance, this is the case for SEMG1, prolactin-induced protein (PIP) and A-kinase anchoring protein 4 (AKAP4), although the experiments were performed by the same team and with the same method [33,34]. The role of SEMG1 in sperm function has been described above, in cases of obesity. The exact role of PIP in sperm is still to be elucidated. However, it has been proposed that it could influence sperm viscosity [100]. As for AKAP4, it is a major component of the sperm fibrous sheath and its absence causes low progressive motility and infertility [101]. Keratin type II cytoskeletal 5 (KRT5) was highlighted in two independent studies, also with opposite variations [32,38]. However, this protein is a common contaminant in mass spectrometry analyses [102] and we believe that it should not be considered as a deregulated sperm protein. Outer dense fibre protein 2 (ODF2) and Tubulin beta-4B chain (TUBB4B) are important cytoskeletal structures of the sperm tail [103,104]. Their decrease in the diabetic individuals could be responsible for the abnormal sperm morphology and motility observed in the investigated samples [32,34]. Serum amyloid P-component (APCS) was more abundant in the spermatozoa from diabetic men [34,38]. The concentration of this protein of a yet unknown function in the seminal plasma has been shown to be positively correlated to the sperm concentration [105]. However, this was not corroborated in the study of An et al. [38], who observed a decrease in the sperm concentration in the diabetic individuals. Ras-related protein Rab-2A (RAB2A) presented increased abundance in the spermatozoa from diabetic men [38] and rats [32]. Although the function of this protein was not studied in human sperm, it has been shown to be involved in acrosome biogenesis in bull [106] and to present increased expression in low-litter size boar spermatozoa [107]. Finally, among the common deregulated proteins was the mitochondrial ATP synthase subunit beta (ATP5B), which was present in either higher [38] or lower [32] abundance in the sperm from the diabetic group. This enzyme synthesises ATP during oxidative phosphorylation, which is used to sustain sperm motility [108].

GO analysis identified enriched biological processes related to several types of metabolic processes, and to tissue homeostasis, regulation of apoptosis, cytoskeleton, fertilisation, and platelet degranulation in humans (Figure 2B, Table S3). In rodent, only two biological processes, glucose metabolic process and response to hydrogen peroxide, were significantly enriched in the short list of deregulated proteins obtained from a single study [32] (Figure 2, Table S3). This suggests disturbances in energy production and the potential involvement of oxidative stress in diabetic rats. 

### 4.3. Tobacco Smoking

A WHO report estimates the number of smokers in the world at 1.3 billion. This corresponds to about 20% of the world’s population and 37% of males aged 15 years or older [109,110]. In addition to its already well-known negative health effects, tobacco smoking negatively affects sperm capacitation, DNA integrity, motility, morphology, numeration, vitality, and semen volume. Different mechanisms have been proposed to explain the impact of smoke components on sperm: increased oxidative stress and inflammation, and alteration of testicular endocrine function, spermatogenesis, and of the hypothalamic–pituitary axis [111,112,113,114]. 

To the best of our knowledge, only one study evaluated the effect of tobacco smoking in humans by using a global proteomic approach [40]. These authors compared the proteomic profile of the seminal plasma of 20 non-smoking normospermic men and 20 smoking patients (smoking at least 10 cigarettes/day), excluding men with clinical history that may cause testicular alterations (Table 1). Using label-free LC-MS/MS, they identified 25 deregulated proteins: 1 absent, 18 less abundant, and 6 more abundant in smokers (Table S1). Functional analysis showed the enrichment of proteins involved in immune and inflammatory responses in smokers [40]. In particular, protein S100A9, a biomarker of inflammation [115], was shown to be able to predict the smoker group [40]. The authors proposed that inflammation of accessory glands and testis could be responsible for the decreased mitochondrial activity and acrosome integrity as well as increased DNA fragmentation they observed in the spermatozoa from smokers [40].

Another proteomic profiling study was performed on mice exposed daily to cigarette smoke. Chen et al. [39] analysed spermatozoa purified from the semen of control and exposed mice by the swim-up method. By 2D-PAGE and MALDI-TOF-MS, they identified 22 differentially expressed proteins; 10 were up-regulated and 12 were down-regulated in exposed mice (Table 1 and Table S1). The variation in abundance of four out of five selected proteins was validated by Western blot analyses [39]. However, the same trend was not observed in Western blot analyses on spermatozoa from smoking men, highlighting a potential limitation in extrapolating data acquired in rodent models to human, possibly because of different testing conditions [39]. GO analysis of the rodent deregulated proteins classified them mainly in cellular, metabolic, and developmental processes; in biological regulation; and in localisation [39].

Several studies analysed the expression level or the activity of sperm proteins involved in different cellular responses in smoking men (Table S1). Cigarette smoking has been shown to decrease the activity of the anti-oxidant enzymes superoxide dismutase (SOD), catalase (CAT), and glutathione-S-transferase (GST) in seminal plasma [116,117,118,119,120]. Interestingly, the only study carried out on purified spermatozoa showed the opposite, with a measured increase in CAT, SOD, and glutathione reductase (GR) activity in smokers [121]. Three studies reported the decrease in the activity of acrosin (ACR), a protease found in the sperm acrosome, in smoking men [57,62,122]. This decrease was associated with a lower inducibility of the acrosome reaction [122]. Kumosani et al. [63] observed a decreased activity of the plasma membrane Ca^2+^-ATPase (ATP2B4) in spermatozoa from smoking men, associated with increased cadmium concentrations in the seminal plasma. They suggested this effect could be responsible for the decreased motility observed in spermatozoa from smokers [63]. A decrease in the expression level of checkpoint kinase 1 (CHK1), an enzyme involved in DNA repair and cell cycle control, has also been reported in spermatozoa from smokers [57]. As the authors also observed an increased sperm DNA fragmentation, they proposed that in smokers, a decline in CHK1 expression could lead to decreased sperm DNA repair [57]. Ghaffari and Rostami [64] observed a negative correlation between duration of smoking and sperm motility and creatine kinase (CK) activity in smoker semen. As CK is an enzyme involved in ATP regeneration, the authors speculated that its decreased content could explain the reduced sperm motility observed in smokers through an impair of sperm energy homeostasis. However, although there was a positive relation between CK and sperm motility, it was not significant [64]. Finally, an increase in two apoptosis markers, tumour necrosis factor receptor superfamily member 6 (Fas) and caspase 3 (CASP3) [55], was also reported in the sperm of smoking men.

After compiling all the proteins deregulated in smoking individuals and applying our criteria on the fold changes, we obtained a list of 54 proteins (Table S2). Three of them—acrosin (ACR), superoxide dismutase (SOD), and catalase (CAT)—were found in three, four, and two independent studies, respectively (Table S2). However, their presence in the list resulted from targeted identification [57,62,116,118,119,121,122] and not global proteomic studies. ACR is an acrosomal enzyme involved in the acrosome reaction and the zona pellucida penetration [123]. It has been proposed as a fertility marker because men with unexplained infertility presented lower acrosine activity than fertile men [124,125]. Its lower activity in the spermatozoa from smoking men could therefore explain fertility issues related to tobacco smoking. Regarding the antioxidant enzymes SOD and CAT, the measured decrease in their activity in the seminal plasma from smoking men [116,118,119] could lead to sperm damages through lipid peroxidation [126].

In humans, GO analysis revealed the significant enrichment of processes involved in response to drug, immune response, apoptosis, oxidative stress, and cell differentiation (Figure 2C, Table S3). In the proteins identified in mice, by a single study [39], processes involved in oxidation–reduction, energy metabolism, and cytoskeleton were significantly enriched (Figure 2C, Table S3). Oxidative stress in sperm seems to be an important process related to smoking in both humans and rodents, as shown by several studies [116,117,118,119,120,121,127].

### 4.4. Exposure to Bisphenol-A

The European Commission and the U.S. Environmental Protection Agency (EPA) define endocrine disruptors as chemical compounds that can interfere with the hormonal system and have negative effects on developmental formation, reproduction, and the immune and nervous system of an individual [128,129]. These compounds can be of natural origin and found in food or be synthesised. The impacts of many endocrine disruptors, such as phthalates, polychlorinated biphenyls, dioxins, pesticides, and parabens, have been studied on male fertility [130,131]. Although there are many endocrine disruptors, bisphenol A (BPA) has been primarily targeted in proteomic studies [41,42,43]. Humans are constantly exposed to BPA. Indeed, these chemical substances are used in the composition of many materials containing food, such as cans and metal lids for epoxy resins and household devices, plastic bottles, and plastic wrapping for polycarbonate plastics [132,133,134]. It has been observed that sperm quality is impacted by BPA exposure. A decrease in sperm motility, vitality, spermatozoa concentration, ATP production, as well as in DNA and acrosome integrity has been shown [41,42,43,135,136,137]. In addition, BPA induces negative effects on the male reproductive tract, causing disturbances in spermatogenesis and testicular formation [42,138,139,140].

In order to understand the impact of BPA on the sperm proteome, Rahman et al. [135] exposed mouse sperm to different concentrations of BPA for six hours. Then, they isolated the motile spermatozoa by the swim-up method from both control and treated groups and monitored the abundance of five fertility-related proteins by Western blotting. They showed that high BPA concentrations (100 µM) induced a down-regulation of ß-actin (ACTB) and an up-regulation of peroxiredoxin-5 (PRDX5), glutathione peroxidase (GPX4), GAPDHS, and succinate dehydrogenase (SDHB) [135] (Table S1). The year after, using the same experimental conditions, the researchers compared the proteome profile from the exposed and non-exposed spermatozoa. Using 2D-PAGE combined with ESI-TOF-MS, they identified 23 differentially expressed proteins, of which eight were more abundant and 15 were less abundant in spermatozoa exposed to high concentrations (100 µM) of BPA (Table 1 and Table S1). Their MS results were validated by Western blotting on six selected proteins [41]. Using GO annotation, they showed that the differentially expressed proteins were involved in energy metabolism or ROS metabolism, or corresponded to structural or fertility related proteins [41].

Later, the same team investigated the effect of gestational BPA oral exposure on the proteomic profile of spermatozoa from the first generation (F1) of male mice [42] (Table 1). They identified two up-regulated proteins and four down-regulated proteins in spermatozoa from males generated by females exposed to 50 mg/Kg of BPA per day, defined as the lowest observed adverse effect level (LOAEL) by the U.S. Environmental Protection Agency (EPA) [141] (Table S1). Of these proteins, five were even significantly deregulated in males generated from females exposed to 5 mg/Kg of BPA per day, defined as the no observed adverse effect level (NOAEL) [42]. Based on a literature search, the authors showed that these five proteins may be involved in oxidative stress response, sperm motility and ATP production [42]. The results were validated in Western blotting for two of the proteins [42]. The same experiment was reproduced to investigate the effects on spermatozoa capacitated before the proteomic analysis [43] (Table 1). The authors identified 15 deregulated proteins in the capacitated spermatozoa from males generated by females exposed to LOAEL doses of BPA compared with males generated from non-exposed females: four were more abundant and 11 were less abundant (Table S1). Four of these proteins were not deregulated in males generated from females exposed to NOAEL doses [43]. Functional annotation revealed that most of the deregulated proteins were associated with cellular energy metabolism, while the others were structural, fertility-related, or stress response proteins [43]. The variation of four selected representative proteins was validated in Western blotting [43]. Recently, Rahman and co-authors extended their study to the second (F2) and third (F3) generations of male mice generated by females exposed to LOAEL doses of BPA during their pregnancy [142]. They specifically focused on the six deregulated proteins identified in their 2017 study [42]. Except for superoxide dismutase (SOD), which was deregulated in spermatozoa from F1 males only, the five other proteins—GPX4, ATP synthase subunit O (ATP5O), glutathione S-transferase (GSTM5), NADH dehydrogenase [ubiquinone] 1 alpha subcomplex subunit 10 (NDUFA10), and Isoaspartyl peptidase/L-asparaginase (ASRGL1)—were also deregulated in spermatozoa from F2 males. All the proteins returned to the control level in F3 males [142]. In addition, Rahman et al. [143] showed that the spermatozoa from male mice (F0) exposed to NOAEL and LOAEL doses of BPA presented increased levels of lactate dehydrogenase (LDH), while control levels of this enzyme were measured for males (F1–F3) generated after breeding with non-exposed females (Table S1).

To the best of our knowledge, in humans, only one study focused on the impact of BPA on sperm proteins. Barbonetti et al. [65] showed that the number of spermatozoa with activated caspase-3 (CASP3) and caspase-9 (CASP9), two apoptosis markers, considerably increased after a 4 h exposure to 300 µM BPA (Table S1). This was associated with an increase in ROS production and a decrease in the mitochondrial membrane potential, demonstrating the role of BPA in the activation of the mitochondrial apoptosis pathway leading to alteration of sperm motility and loss of DNA integrity [65].

By compiling these studies, we obtained a list of 36 proteins which seem to be deregulated in relation to BPA exposure (Table S2). Fourteen of them (39%) are common to at least two of the cited studies, which is explained by the fact that they were identified by the same research team with the same experimental strategy [41,42,43,135,143]. However, for some of them, opposite variations were observed according to the studies. Indeed, the samples investigated were either spermatozoa directly exposed to BPA [41,135], or spermatozoa from F1 males generated from females exposed to BPA during pregnancy [42,43]. In the case of exposed spermatozoa, the effects of BPA could obviously not be attributed to a disturbance in spermatogenesis. Therefore, decreased abundance was attributed to degradation of the proteins, while increased abundance (e.g., for GAPDHS) was attributed to post-translational modification of the protein [41,135]. Four sperm structural proteins were deregulated following BPA exposure: ACTB, ROPN1, FABP9, and ODF2 [41,43,135]. Altered expression of these proteins has been shown to cause alteration in sperm morphology and/or motility [103,144,145,146]. The decrease in ATP5O, an enzyme involved in oxidative phosphorylation, has been proposed to be responsible for the reduced sperm ATP levels and motility observed after BPA exposure [41,42,43]. The deregulation of four antioxidant enzymes, GPX4, PRDX5, GSTM5, and SOD2, was also observed following BPA exposure. As proposed for tobacco smoking, such deregulation in the antioxidant defence system could lead to sperm oxidative damage [11,92]. Similarly, it has been shown that a decrease in the expression of Prohibitin (PHB), as observed after BPA exposure [41,43], causes an increase in ROS levels and thereby a decrease in sperm motility [147].

We performed the GO analysis on the list of mouse proteins only, as only two proteins were highlighted in humans. This analysis showed a significant enrichment of processes related to oxidation–reduction processes, energy production, metabolism, and response to drug (Figure 2D, Table S3).

## 5. Conclusions

Over the past 30 years, an increasing number of studies have demonstrated the negative impact of environmental and lifestyle factors on male fertility. In particular, smoking, diabetes, psychological stress, drug or alcohol use, endocrine disruptors, and nutrition have been shown to have a deleterious effect on sperm parameters (numeration, motility, morphology, vitality), as well as on DNA integrity, capacitation, and ATP production by mitochondria [22,24,148]. Here, we reviewed the literature on the effect of obesity, diabetes, smoking, and BPA exposure on sperm proteomics. These proteomic data provide information for the understanding of the molecular mechanisms observed in male fertility disturbances in response to these factors. Among the proteins highlighted in the different studies, many are common to several risk factors (Table S2) and should therefore be considered for further analysis. Some of them present known functions in male fertility (Table 2), as in the case of ODF2, which was deregulated for each risk factor, and of ODF1, GAPDHS, and RAB2A, which were deregulated in the case of three risk factors. These proteins could become new diagnostic markers for male infertility and their targeted identification should be considered in addition to routine sperm analysis. Indeed, variation in protein abundances were observed even in cases of normospermic samples (e.g., [33,37,40], showing that routine sperm analyses are not sufficient to identify causes of infertility.

## Figures and Tables

**Table 1:** Studies using global proteomic analyses to identify sperm deregulated proteins in relation to male infertility risk factors.

Obesity							
References	Species	Investigated Groups	Sperm Preparation	Working Sample	Method for Protein Identification	Method for Protein Quantification	Results *
Ferigolo et al. 2019 [31]	Human	27 obese men (BMI ≥ 33 kg/m^2^), 20 eutrophic men (18.5 kg/m^2^ ≤BMI ≤ 25 kg/m^2^); the samples from 4 men were pooled and 4 pools were obtained per group. No differences in sperm volume, concentration, and progressive motility, but significant differences in non-progressive motility and morphology.	Centrifugation	Seminal plasma	In-solution digestion; LC-MS/MS (hybrid quadrupole-Orbitrap)	Label-free; Maxquant software; Ibaq; all fold changes were considered.	485 proteins identified; 70 differentially expressed proteins: 50 more abundant, 19 less abundant, and 1 exclusive in the obese group.
Carvalho et al. 2021 [32]	Rat	10 rats fed with a control diet and 10 rats fed with a hyperglycidic diet for 266 days. No differences in sperm concentration but differences in sperm morphology between groups.	Washes with centrifugation	Pellet after centrifugation (no purification)	SDS-PAGE, LC-MS/MS (QTOF)	Label-free, calculation of the emPAI.	144 proteins identified; 15 differentially expressed proteins: 8 more abundant and 7 less abundant in obese rats.
Kriegel et al. 2009 [33]	Human	5 normospermic men (mean BMI = 22 kg/m^2^), 2 non-diabetic obese men (mean BMI = 33 kg/m^2^); 3 semen samples for each. No significant differences in sperm parameters between the 2 groups.	50–90% gradient	Progressive spermatozoa	2D-PAGE (DIGE); MALDI-TOF-MS	Fluorescent labelling; image acquisition and identification of differentially expressed proteins with a fluorescence imager. Spots with a fold change set to 2.0 were excised and analysed.	2700 fluorescent protein spots detected; 9 differentially expressed proteins: 2 more abundant, 6 less abundant, and one more or less abundant according to the gel spot, in obese men.
Paasch et al. 2011 [34]	Human	21 normospermic and clinically healthy men (mean BMI = 22.5 kg/m^2^), 13 non-diabetic obese men (mean BMI = 34 kg/m^2^); 3 semen samples for each. Sperm progressive motility and morphology were significantly different between the groups.	50–90% gradient	Progressive spermatozoa	2D-PAGE (DIGE); MALDI-TOF-MS	Fluorescent labelling; image acquisition and identification of differentially expressed proteins with a fluorescence imager. Spots with a fold change ≤ −1.6 or ≥1.6 were excised and analysed.	3187 fluorescent protein spots detected; 7 differentially expressed protein: 6 more abundant and 1 less abundant in obese men.
Liu et al. 2015 [35]	Human	3 normospermic fertile men (mean BMI = 24 kg/m^2^), 3 obese men (mean BMI = 33.5 kg/m^2^) with severe asthenozoospermia (normal concentration and morphology, but progressive motility <20%).	45% Percoll gradient	Spermatozoa purified from seminal plasma	FASP, LC-MS/MS (LTQ-Orbitrap)	Label free; Maxquant software; all fold changes were considered.	1975 proteins identified; 127 differentially expressed proteins: 22 more abundant and 105 less abundant in obesity-associated asthenozoospermia.
Peng et al. 2019 [36]	Mouse	6 mice fed with control diet and 6 mice fed with high-fat diet for 10 weeks. No differences in sperm concentration but significant differences in sperm motility, progressive motility and morphology.	45% Percoll gradient	Spermatozoa purified from seminal plasma	FASP, LC-MS/MS (LTQ-Orbitrap)	Label-free; Maxquant software; only proteins with a fold change of ≥1.3 or ≤0.7 were considered.	1562 proteins identified; 160 differentially expressed proteins: 60 more abundant and 100 less abundant in obese mice.
Pini et al. 2020 [37]	Human	5 men with healthy weight (BMI ≤ 25 kg/m^2^), 5 obese men (BMI ≥ 33 kg/m^2^); all normospermic.	45–90% gradient	Progressive spermatozoa	FASP, LC-MS/MS (hybrid quadrupole-Orbitrap)	Label-free quantification with normalised weighted spectra (NWS); only proteins with a fold change of ≥1.5 or ≤0.5 were considered.	2034 proteins identified; 27 differentially expressed proteins: 3 more abundant and 24 less abundant in obese men.
**Diabetes**							
**References**	**Species**	**Investigated groups**	**Sperm** **Preparation**	**Working** **Sample**	**Method for Protein** **Identification**	**Method for Protein Quantification**	**Results**
Carvalho et al. 2021 [32]	Rat	10 control rats and 5 rats injected with streptozotocin to induce diabetes. No differences in sperm concentration but differences in sperm morphology between groups.	Washes with centrifugation	Pellet after centrifugation (no purification)	SDS-PAGE, LC-MS/MS (QTOF)	Label-free, calculation of the emPAI.	144 proteins identified, 15 differentially expressed proteins: 3 more abundant and 12 less abundant in diabetic men.
Kriegel et al. 2009 [33]	Human	5 normospermic donors, 2 type-1 diabetic patients; 3 semen samples for each. No significant differences in sperm parameters between the 2 groups.	50–90% gradient	Progressive spermatozoa	2D-PAGE (DIGE); MALDI-TOF-MS	Fluorescent labelling; Image acquisition and identification of differentially expressed proteins with a fluorescence imager. Spots with a fold change set to 2.0 were excised and analysed.	2700 fluorescent protein spots detected; 7 differentially expressed proteins: 4 more abundant, 2 less abundant, and one more or less abundant according to the gel spot, in diabetic men.
Paasch et al. 2011 [34]	Human	21 normospermic and clinically healthy individuals, 8 type-1 diabetic individuals, and 7 type-2 diabetic individuals; 3 semen samples for each. Sperm progressive motility and morphology were significantly different between the groups.	50–90% gradient	Progressive spermatozoa	2D-PAGE (DIGE); MALDI-TOF-MS	Fluorescent labelling; Image acquisition and identification of differentially expressed proteins with a fluorescence imager. Spots with a fold change ≤−1.6 or ≥1.6 were excised and analysed.	3187 fluorescent protein spots detected; 8 differentially expressed protein in type-1 diabetic patients: 6 more abundant and 2 less abundant; 39 differentially expressed proteins in type-2 diabetic patients: 12 more abundant, 25 less abundant, and 2 more or less abundant according to the gel spot.
An et al. 2018 [38]	Human	6 healthy men and 6 type-2 diabetic men. Sperm volume and concentration were significantly different between the groups.	Centrifugation	Pellet after centrifugation (no purification)	Gel-free digestion; LC-MS/MS (hybrid quadrupole-Orbitrap)	Itraq labelling; only proteins with a fold change of >1.2 or <0.83 were considered.	1114 proteins identified; 357 differentially expressed: 38 less abundant and 319 more abundant in diabetic men.
**Tobacco smoking**							
**References**	**Species**	**Investigated groups**	**Sperm** **Preparation**	**Working** **Sample**	**Method for Protein** **Identification**	**Method for Protein Quantification**	**Results**
Chen et al. 2015 [39]	Mouse	3 mice exposed daily to cigarette smoke for 2 weeks, and 3 non-exposed mice. No differences in sperm motility between the groups.	Swim up	Spermatozoa	2D-PAGE, MALDI-TOF-MS	Image acquisition and spot density analysis. Spots with a fold change set to 2.0 were excised and analysed.	More than 1000 protein spots detected; 22 differentially expressed proteins: 10 more abundant and 12 less abundant proteins in exposed mice.
Antoniassi et al. 2016 [40]	Human	20 non-smoking normospermic men and 20 smoking patients (≥ 10 cigarettes/day). The samples were distributed into 4 pools per group. No significant differences in sperm parameters between the 2 groups.	Centrifugation	Seminal plasma	In-solution digestion; LC-MS/MS (hybrid quadrupole-Orbitrap)	Label-free; Maxquant software; Ibaq; all fold changes were considered.	422 proteins identified, 25 differentially expressed proteins: 1 absent, 6 more abundant, and 18 less abundant in smokers.
**Bisphenol-A exposure**							
**References**	**Species**	**Investigated groups**	**Sperm** **Preparation**	**Working** **Sample**	**Method for Protein** **Identification**	**Method for Protein Quantification**	**Results**
Rahman et al. 2016 [41]	Mouse	Spermatozoa from 3 mice were exposed or not to various concentrations (0.0001–100 µM) of BPA.	Swim up	Spermatozoa	2D-PAGE, ESI-QTOF	Image acquisition and spot density analysis. Spots with significant density changes were excised and analysed.	399 spots detected; 23 differentially expressed proteins: 8 more abundant and 15 less abundant in spermatozoa exposed to the upper concentration of BPA.
Rahman et al. 2017 [42]	Mouse	Male mice (F1; n = 3) generated from females which were orally exposed or not to various concentrations (0.05–50 mg/kg per day) of BPA during pregnancy.	Swim up	Spermatozoa	2D-PAGE, ESI-QTOF	Image acquisition and spot density analysis. Spots with significant density changes were excised and analysed.	284 spots detected, 6 differentially expressed proteins: 2 more abundant and 4 less abundant in males generated from females exposed to BPA.
Rahman et al. 2018 [43]	Mouse	Male mice (F1; n = 3) generated from females which were orally exposed or not to various concentrations (0.05–50 mg/kg per day) of BPA during pregnancy.	Capacitation, wash and swim up	Capacitated spermatozoa	2D-PAGE, ESI-QTOF	Image acquisition and spot density analysis. Spots with significant density changes were excised and analysed.	285 spots detected, 15 differentially expressed proteins: 4 more abundant and 11 less abundant in males generated from females exposed to the upper concentration of BPA.

* For some studies, the number of proteins differentially expressed presented in this table may be different to the number highlighted in the original articles. This is because (1) we decided to count proteins only once if they appeared in different gel spots; (2) some proteins, although with different names, were shown to possess the same Uniprot accession number; and (3) some data were not provided in the original articles. DIGE: difference gel electrophoresis; empAI: exponentially modified protein abundance index; FASP: filter-assisted sample preparation; fold change: ratio of the amount of a protein in the investigated group to its amount in the control group; iBAQ: intensity-based absolute quantification, in which the protein content is normalised to the total number of potential peptides; iTRAQ: isobaric tag for relative and absolute quantitation.

**Table 2:** Proteins associated with fertility and deregulated in relation to several male infertility risk factors.

Protein (Gene Name)	Risk Factors	Role in Sperm Fertility	References
Acrosin (ACR)	Diabetes, Tobacco smoking	Acrosomal enzyme involved in acrosome reaction and zone pellucida penetration.	[123]
Actin (ACTB)	Obesity, BPA exposure	Cytoskeletal protein, plays a role in sperm motility and the acrosome reaction.	[146,149]
Fructose-bisphosphate aldolase A (ALDOA)	Diabetes, Tobacco smoking	Glycolytic enzyme involved in zone pellucida binding.	[150]
Apolipoprotein A-I (APOA1)	Obesity, Tobacco smoking	Sterol acceptor in the seminal fluid involved in sperm motility activation.	[87,88]
ATP synthase subunit beta (ATP5B)	Obesity, Diabetes	Involved in ATP synthesis during oxidative phosphorylation, which is important to sustain sperm motility.	[108]
Caspase 3 (CASP3)	Tobacco smoking, BPA exposure	Functional enzyme of the apoptosis process. Its high expression is related to athenospermia and teratospermia.	[151,152]
Calicin (CCIN)	Obesity, Diabetes	Cytoskeletal protein of the sperm head.	[153]
Clusterin (CLU)	Obesity, Diabetes	Seminal plasma protein involved in sperm maturation and capacitation.	[154,155]
Epididymal secretory protein E3-beta (EDDM3B)	Obesity, Diabetes	Epididymal protein, may be involved in sperm maturation.	[156]
Glyceraldehyde-3-phosphate dehydrogenase (GAPDHS)	Obesity, Diabetes, BPA exposure	Glycolytic enzyme involved in sperm motility.	[75,83]
Glutathione peroxidase 4 (GPX4)	Tobacco smoking, BPA exposure	Antioxidant enzyme implicated during spermatogenesis and sperm maturation.	[157]
Neutrophil gelatinase-associated lipocalin (LCN2)	Obesity, Tobacco smoking	Modulates sperm capacitation.	[158]
Lactotransferrin (LTF)	Obesity, Diabetes	Bound to eppin in the EPC and involved in protection of spermatozoa and motility regulation.	[34,76]
Outer dense fiber protein 1 (ODF1)	Obesity, Diabetes, Tobacco smoking	Chaperone protein composing the outer dense fibres (ODF) of the sperm tail, involved in sperm structure and motility.	[159]
Outer dense fiber protein 2 (ODF2)	Obesity, Tobacco smoking, diabetes, BPA	Major component of the outer dense fibres (ODF) of the sperm tail, involved in sperm motility.	[103]
Protein/nucleic acid deglycase DJ-1 (PARK7)	Obesity, Diabetes	Oxidative stress response protein involved in fertilisation process and sperm motility.	[160,161]
Pyruvate dehydrogenase E1 component subunit a, testis-specific form, mitochondrial (PDHA2)	Diabetes, Tobacco smoking	Involved in the development of spermatogenic cells.	[162]
Prolactin-inducible protein (PIP)	Obesity, Diabetes	May influence sperm viscosity.	[100]
Phospholipase A2, membrane associated (PLA2B)	Obesity, Tobacco smoking	Involved in membrane fusion events occurring during the acrosome reaction and fertilisation.	[85]
Prostaglandin-H2 D-isomerase (PTGDS)	Obesity, Diabetes	Carrier protein for thyroid hormone and retinoids involved in spermatogenesis and sperm maturation.	[163]
Ras-related protein Rab-2A (RAB2A)	Obesity, Diabetes, BPA exposure	Acrosome biogenesis.	[106]
Semenogelin-1 (SEMG1)	Obesity, Diabetes	Seminal protein which inhibits premature sperm capacitation.	[76]
Alpha-1-antitrypsin (SERPINA1)	Obesity, Diabetes	Serine protease inhibitor involved in the liquefaction cascade of the ejaculated sperm.	[158]
S-phase kinase-associated protein 1 (SKP1)	Obesity, Diabetes	Regulates meiosis during spermatogenesis.	[164]
Sperm surface protein Sp17 (SPA17)	Diabetes, BPA exposure	Fibrous sheath protein involved in the binding to the oocyte zona pellucida.	[165]
Sperm acrosome membrane-associated protein 3 (SPACA3)	Obesity, Diabetes	Acrosomal protein involved in the binding to the oocyte.	[166,167]
Triosephosphate isomerase (TPI1)	Diabetes, BPA exposure	Glycolytic enzyme involved in the binding to the oocyte zone pellucida.	[149]
Tubulin beta chain (TUBB4B)	Obesity, Diabetes	Cytoskeletal protein of the sperm tail.	[104]

## Data Availability

Not applicable.

## Supplementary Materials

The following are available online at https://www.mdpi.com/article/10.3390/ijms222313164/s1.
