# Supplementary material for: Influence of Risk Factors for Male Infertility on Sperm Protein Composition
Source: Int J Mol Sci. 2021 Dec 6;22(23):13164. doi: 10.3390/ijms222313164 (PMC8658491; doi:10.3390/ijms222313164)
Supplement: Supplementary file 1 [file ijms-22-13164-s001.zip › Supplementary information.pdf]

Influence of risk factors for male infertility on sperm protein composition

Supporting information

Marie Bisconti [1], Jean-François Simon [2], Sarah Grassi [1], Baptiste Leroy [3], Baptiste Martinet [4], Vanessa Arcolia [2], Vladimir Isachenko [5,*] and Elise Hennebert [1]

[1] Laboratory of Cell Biology, Research Institute for Biosciences, University of Mons, Place du Parc 20, 7000 Mons, Belgium

[2] Fertility Clinic, CHU Ambroise Paré Hospital, Boulevard Kennedy 2, 7000 Mons, Belgium

[3] Laboratory of Proteomics and Microbiology, CISMa, Research Institute for Biosciences, University of Mons, 7000 Mons, Belgium

[4] Evolutionary Biology & Ecology, Université Libre de Bruxelles, Avenue Paul Héger - CP 160/12, 1000 Brussels, Belgium

[5] Department of Obstetrics and Gynecology, University of Cologne, Kerpener Strasse 34, 50931 Cologne, Germany

***Corresponding author:**

Vladimir Isachenko, Department of Obstetrics and Gynecology, University of Cologne, Kerpener Strasse 34, 50931 Cologne, Germany.

E-mail address: vladimir.isachenko@uk-koeln.de

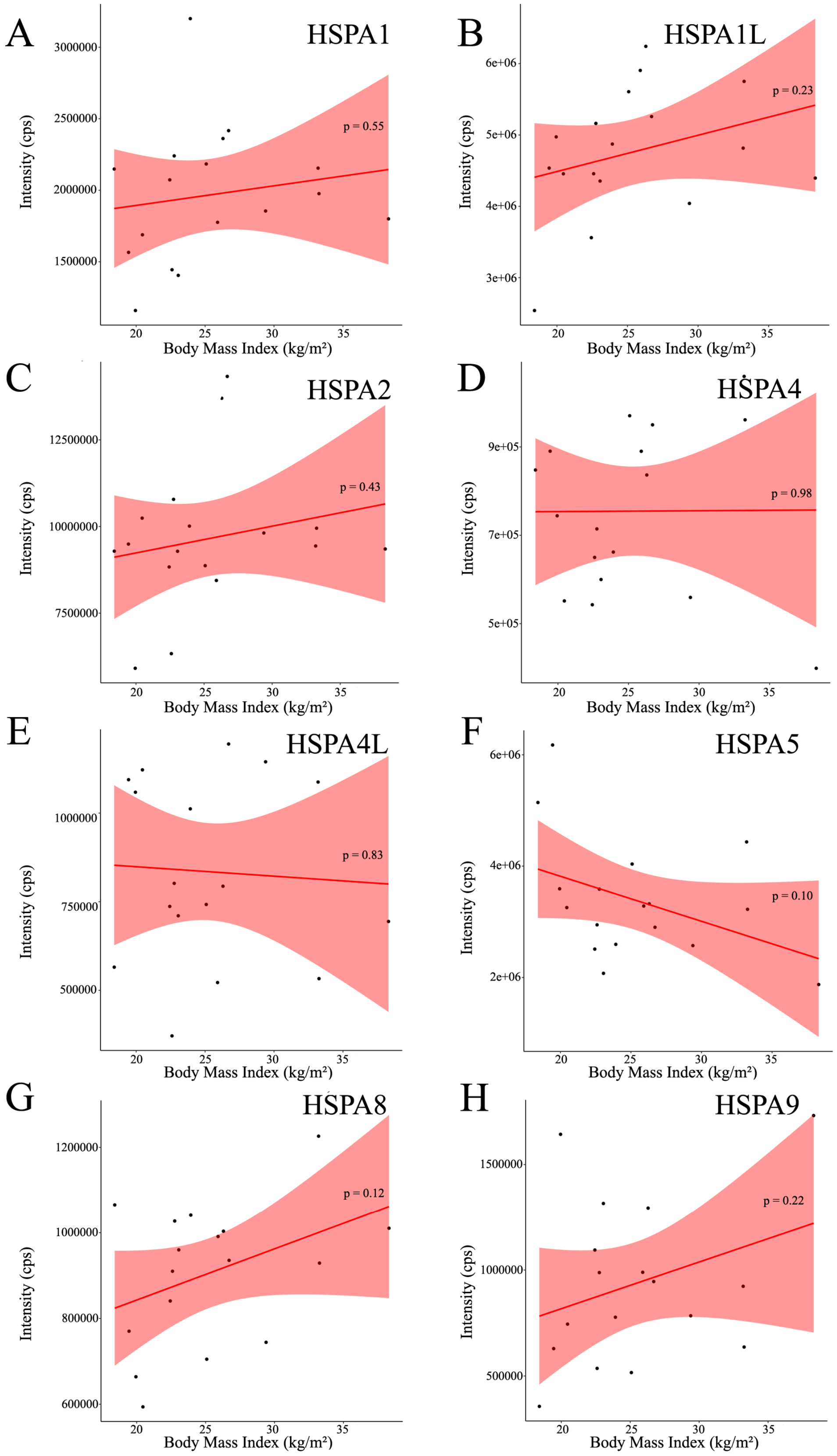

Figure S1. Linear regression analysis between intensity of each isoform of HSP70 and BMI. The analysis was carried out on 17 normospermic individuals with BMI ranging between 18.4 and 38.3 Kg/m^2. The abundance of the 8 HSP70 isoforms was obtained by LC-MRM and normalized to aconitate hydratase, used as loading control based on its apparent stability in spermatozoa (Castillo et al., 2019). Red lines represent the linear fit of data. Red areas represent Confidence Interval at 95%.

Supplementary reference:

Castillo, J., Bogle, O. A., Jodar, M., Torabi, F., Delgado-Dueñas, D., Estanyol, J. M., Ballescà, J.L., Miller, D. & Oliva, R. (2019). Proteomic Changes in Human Sperm During Sequential in vitro Capacitation and Acrosome Reaction. Frontiers in Cell and Developmental Biology, 7, 295.
